# Effect of Chitooligosaccharides on TLR2/NF-κB Signaling in LPS-Stimulated RAW 264.7 Macrophages

**DOI:** 10.3390/molecules30102226

**Published:** 2025-05-20

**Authors:** Mengting Zhao, Shurong Pang, Yiqing Gao, Ting Li, Hongrui Jiang

**Affiliations:** 1 College of Light Industry and Food Engineering, Guangxi University, Nanning 530004, China; mtzhao2025@163.com (M.Z.); 18378065353@163.com (S.P.); 18335448576@163.com (Y.G.); 18376168239@163.com (T.L.); 2 Key Laboratory of Deep Processing and Safety Control for Specialty Agricultural Products in Guangxi Universities, Education Department of Guangxi, Nanning 530004, China

**Keywords:** degree of polymerization, chitooligosaccharides, TLR2, anti-inflammatory, NF-κB signaling pathway

## Abstract

Chitooligosaccharides (COSs), degraded products of chitosan or chitin, are attracting growing interest owing to their low degree of polymerization (DP), high solubility, and prominent anti-inflammatory activity. However, the correlation between their structure and anti-inflammatory activities still needs to be explored. In this study, we use LPS-stimulated RAW 264.7 macrophages as an inflammatory model to systematically evaluate COS1–7 for their effects on inflammatory mediators and NF-κB signaling pathways. The results of Griess assay, ELISA, and real-time quantitative PCR show that COSs can inhibit the expression of NO, iNOS, and pro-inflammatory cytokines (IL-6, TNF-α, MCP-1 and IL-1β), thereby attenuating inflammatory signaling. Notably, chitohexaose (COS6) exhibits the most significant anti-inflammatory effect, reducing the mRNA levels of LPS-induced iNOS, IL-6, and IL-1β and the production of IL-6 and TNF-α by more than 50%. Transcriptome, western blotting, and real-time quantitative PCR analysis reveal that COSs can inhibit the activation of the NF-κB signal pathway by down-regulating TLR2 levels. Additionally, molecular docking confirms that COSs retard TLR2/4 dimerization and LPS recognition by TLR4, affecting downstream signaling cascades. In summary, this study provides a valuable insight into the potential anti-inflammatory mechanism of COSs and highlights the possible applications in human health promotion by modulating receptor-mediated signaling pathways.

## 1. Introduction

Inflammation serves as a vital defense mechanism of the body against external stimuli, mediated by the secretion of nitric oxide (NO) and pro-inflammatory cytokines [1]. Nevertheless, excessive inflammation can contribute to the development of chronic diseases, including asthma [2], rheumatoid arthritis [3], atherosclerosis [4], inflammatory bowel disease [5], and cancer [6]. Current anti-inflammatory treatments, including both steroidal and non-steroidal medications, are effective but are often associated with undesirable side effects [1,7]. Therefore, there is an urgent need for safer alternatives, especially bioactive compounds derived from food sources, that can mitigate inflammation while supporting overall health.

Chitosan is predominantly obtained by deacetylation of chitin, which is extracted from the exoskeletons of crustaceans such as shrimp and crab and has been widely used as a preservative and additive in food processing [8]. Chitooligosaccharides (COSs), produced by degrading chitosan through enzymatic or chemical hydrolysis [9], consist of β-(1→4)-linked N-acetylglucosamine and deacetylated glucosamine units, with a degree of polymerization (DP) of less than 20 [10]. COSs exhibited better solubility and bioactivity compared to chitosan. COSs have been found to exhibit beneficial biological activities, including anti-tumor [11], antibacterial [12], antioxidant [13], and immunomodulatory effects [14], which makes them valuable in the field of food and agriculture. The anti-inflammatory properties of COSs have recently attracted considerable scientific interest. Notably, studies have demonstrated that three structural parameters, DP, degree of acetylation (DA), and molecular weight (MW), critically influence the anti-inflammatory efficacy of COSs [10,15]. The results show that low molecular weight COSs can effectively inhibit lipopolysaccharide (LPS)-stimulated pro-inflammatory factors in murine microglia BV2 cell, including the expression of NO, inducible nitric oxide synthase (iNOS), cyclooxygenase-2 (COX-2), tumor necrosis factor α(TNF-α), interleukin-6 (IL-6), and interleukin-1β (IL-1β) [16]. Similarly, COS mixtures with DA ranging from 0% to 85% have shown significant anti-inflammatory effects in LPS-stimulated RAW 264.7 macrophages, with a DA of 12% exhibiting the strongest inhibitory effect on overexpression of iNOS, TNF-α, and IL-6 via the NF-κB signaling pathway [17]. Additionally, other studies have investigated the effect of DP on the anti-inflammatory properties of COSs. It is noteworthy that the anti-inflammatory activity of COSs appears to depend on DP. Some studies have reported that certain COS mixtures can enhance inflammatory signaling. For example, a COS mixture (DP 1–5) derived from commercial shrimp chitin suspension enhanced MAPK phosphorylation, NF-κB activation, and iNOS expression, thereby increasing the production of NO, TNF-α, and IL-6 in LPS-stimulated RAW 264.7 macrophages [18]. Conversely, some COS mixtures exhibit potent anti-inflammatory activities. For instance, COS mixtures with low DP (DP < 8) showed a greater reduction in NO production compared to those with higher DP (8 < DP < 16) [19]. A COS mixture (DP 2–6) was found to inhibit LPS-stimulated overexpression of IL-6 and TNF-α in RAW 264.7 macrophages by modulating the MAPK and PI3K/Akt signaling pathways [20] while also suppressing the LPS-stimulated overexpression of IL-1β and NO through the MAPK and NF-κB signaling pathways [21]. These findings demonstrate that the structural parameters of COSs, particularly DP, significantly influence their effects on inflammatory pathways and downstream factors. Notably, variations in DP can even lead to opposing effects [18]. The differences in the inflammatory effects of COS mixtures may be attributed to the composition and DP of the COS mixtures, which significantly influence their activities. Different COS mixtures may contain monomers and oligomers with varying DPs, which can affect their interactions with downstream signaling pathways. However, existing studies predominantly focus on COS mixtures, leaving a limited understanding of the structure–activity relationships, especially regarding COSs with DP, i.e., from 1 (COS1) to 7 (COS7).

Lipopolysaccharide (LPS), a crucial component of the Gram-negative bacteria, is primarily composed of lipids and polysaccharides and serves as a key trigger of the immune response in mammals [22]. LPS can induce robust inflammatory responses, such as sepsis [23], atherosclerosis [24], and obesity [25]. The biological activity of LPS is mainly attributed to its lipid A moiety. When it is recognized and bound by lipopolysaccharide-binding protein (LBP) and cluster of differentiation 14 (CD14), the complex primarily interacts with Toll-like receptor 4 (TLR4), leading to the activation of intracellular signaling pathways. This process triggers the nuclear translocation of the transcription factor NF-κB, which, in turn, promotes the expression of various pro-inflammatory cytokines, thereby triggering a cascade of inflammatory responses [26]. Toll-like receptors (TLRs) are type I transmembrane proteins that interact with LPS and are responsible for signal transduction in the innate immune system [27]. TLR4, in particular, has been identified as a primary receptor for LPS recognition in macrophages [28]. Additionally, TLR2 has been shown to mediate LPS-induced signaling [29]. After being stimulated by LPS, TLR4 and TLR2 are activated and undergo dimerization [28,30,31], initiating downstream signaling pathways such as NF-κB [32,33], MAPK [34], and STAT3 [31]. The pathways promote the transcription and expression of inflammatory factors, thereby stimulating inflammatory signaling [35]. Previous studies suggest that COSs regulate immune cells by binding with TLR4, influencing the immune response to LPS stimulation. Some COS mixtures have been shown to enhance inflammatory signaling by interacting with TLR4. It has been proposed that COSs may interact with TLRs in LPS-stimulated macrophages, regulating inflammation mainly though signaling pathways like NF-κB and MAPK. For instance, a COS mixture (DP 1–5) enhanced MAPK signaling phosphorylation, activated NF-κB, induced iNOS expression, and increased the production of NO, TNF-α, and IL-6 by binding to TLR4 in LPS-stimulated RAW 264.7 macrophages [18]. Conversely, other COS mixtures have exhibited potent anti-inflammatory activities. These COS compounds can regulate the expression of TLR4 and inhibit the activation of the NF-κB and MAPK signaling pathways. It was observed that a COS mixture (DP 2–6) significantly upregulated TLR4 and TLR5 expression in LPS-stimulated RAW 264.7 macrophages while inhibiting the phosphorylation of NF-κB and MAPK [36]. Despite these findings, the interaction between COS and TLR2 in the regulation of LPS-stimulated macrophage inflammatory signaling remains less explored. The effects of the DP of COSs on receptor interactions and their distinct impact on LPS-stimulated inflammatory signaling in RAW 264.7 macrophages are still not fully understood.

In this study, COS1–7 were selected to evaluate the structure–activity relationship between polymerization degree and anti-inflammatory activity in LPS-stimulated macrophages. The anti-inflammatory effects of COSs were comprehensively assessed through multiple experimental approaches, including quantification of NO release, profiling of cytokine levels, and pro-inflammatory gene transcription. To investigate the underlying molecular mechanisms, RNA sequencing (RNA-Seq), western blotting, and molecular docking were utilized to determine how DP modulates COS activities in LPS-induced inflammatory signaling.

## 2. Results

### 2.1. Effect of COS on the Viability of RAW 264.7 Cells

**Figure 1:**
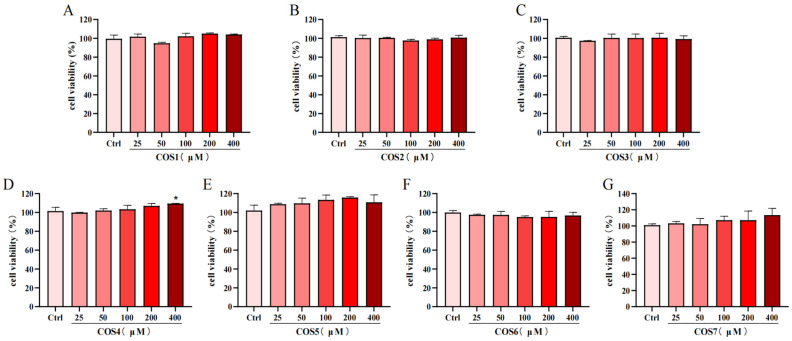
Effects of various concentrations of chitooligosaccharides (0–400 μM) on the viability of RAW264.7 cells. (**A**) Glucosamine (COS1), (**B**) Chitobiose (COS2), (**C**) Chitotriose (COS3), (**D**) Chitotetraose (COS4), (**E**) Chitopentaose (COS5), (**F**) Chitohexaose (COS6), and (**G**) Chitoheptaose (COS7), with results expressed as a percentage of cell viability relative to the Control (Ctrl) group. Compared to the Ctrl group, * *p* < 0.05.

COS1–7 did not inhibit cell viability at concentrations up to 400 μM (Figure 1). Notably, COS4 at 400 μM significantly (*p* < 0.05) increased cell viability but had no significant effect (*p* >0.05) within a concentration range of 25 μM to 200 μM (Figure 1D). This finding was consistent with a prior study, which showed that COSs with a degree of polymerization of 2–6 exerted little to no toxicity on RAW 264.7 macrophages and, at a 600 μM concentration, even promoted cell growth [37]. Given that COSs with all seven polymerization degrees showed no significant effects on the viability of RAW 264.7 macrophages at 200 μM, this concentration was chosen in subsequent experiments.

### 2.2. Effect of COS Polymerization Degree on LPS-Induced Inflammatory Signaling

**Figure 2:**
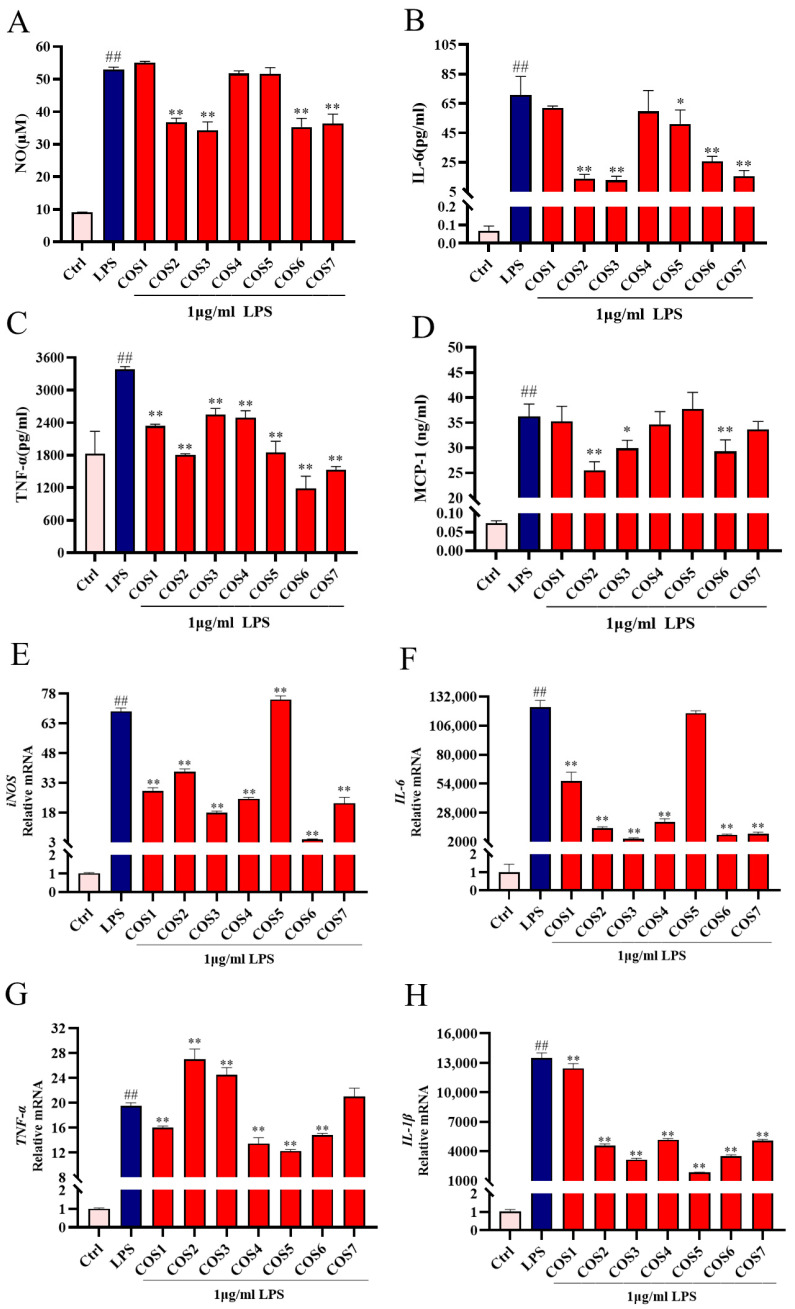
Effect of COS1–7 on the expression of NO, iNOS, and cytokines (IL-6, TNF-α, MCP-1) in LPS-stimulated cells. NO release (**A**) was detected by Griess assay kit. ELISA was employed to detect the protein expression levels of chitooligosaccharides with different DP for (**B**) IL-6 and (**C**) TNF-α and (**D**) MCP-1 in cells. RT-qPCR was utilized to access the mRNA gene levels of (**E**) iNOS, (**F**) IL-6, (**G**) TNF-α, as well as (**H**) IL-1β. ## *p* < 0.01 relative to the Ctrl group, * *p* < 0.05 and ** *p* < 0.01 relative to the LPS group. In all the figures of this paper, the error bars represent the standard deviation.

To evaluate the impact of COS1–7 on LPS-induced inflammatory signaling, we analyzed the levels of NO and pro-inflammatory cytokines (TNF-α, IL-6, and MCP-1) secreted by RAW 264.7 macrophages (Figure 2A–D). Compared to the Ctrl group, LPS stimulation significantly increased the levels of NO, IL-6, TNF-α, and MCP-1 (*p* < 0.01). The levels of NO, IL-6, TNF-α, and MCP-1 in the LPS-treated group were 53.04 μM, 70.78 pg/mL, 3383.73 pg/mL, and 36.22 ng/mL, respectively. Pretreatment with COS2, COS3, COS6, and COS7 significantly inhibited LPS-induced NO production (*p* < 0.01) (Figure 2A). Similarly, COS2, COS3, COS5, COS6, and COS7 significantly reduced IL-6 secretion (*p* < 0.05) (Figure 2B). COS2, COS3, and COS6 effectively inhibited MCP-1 secretion (Figure 2D). Additionally, pretreatment with all COSs significantly suppressed TNF-α secretion, with COS6 showing the strongest inhibitory property (*p* < 0.01) (Figure 2C). Overall, COS2 and COS6 exhibited significant reductions in LPS-induced NO release and proinflammatory cytokine expression (Figure 2A–D). Specifically, in the COS2-treated group, the concentrations of NO, IL-6, TNF-α, and MCP-1 were, respectively, 36.78 μM, 13.95 pg/mL, 1805.82 pg/mL, and 25.54 ng/mL, while in the COS6-treated group, they were 35.29 μM, 25.72 pg/mL, 1183.73 pg/mL, and 29.28 ng/mL, respectively.

RT-qPCR analysis of the mRNA expression levels of iNOS and cytokines (IL-6, TNF-α and IL-1β) revealed that COS1–7 inhibited LPS-induced mRNA expression to varying extents (Figure 2E–H). Compared with the Ctrl group, LPS stimulation resulted in a 69-fold increase in the upregulation of iNOS, a 122,394-fold increase in IL-6, a 20-fold increase in TNF-α, and a 13,470-fold increase in IL-1β. Except COS5, other COSs significantly reduced iNOS (Figure 2E) and IL-6 (Figure 2F) mRNA levels (*p* < 0.01). COS3 and COS6 showed the most pronounced effects. Additionally, COS1, COS4, COS5, and COS6 significantly inhibited TNF-α mRNA overexpression (*p* < 0.01) (Figure 2G). All COSs significantly reduced IL-1β mRNA levels (*p* < 0.01) (Figure 2H). Notably, COS6 lowered the relative levels of iNOS, IL-6, TNF-α, and IL-1β in LPS-stimulated cells to 4, 8278, 15, and 3502, respectively, compared to the Ctrl group, exhibiting the most stable and potent inhibitory effect on the transcriptional expression of iNOS and pro-inflammatory cytokines (Figure 2E–H). Given its superior anti-inflammatory properties, COS6 was selected for subsequent RNA-seq analysis to elucidate the underlying molecular mechanisms.

### 2.3. Effect of COS6 on Transcriptome of LPS-Stimulated RAW 264.7 Macrophages

**Figure 3:**
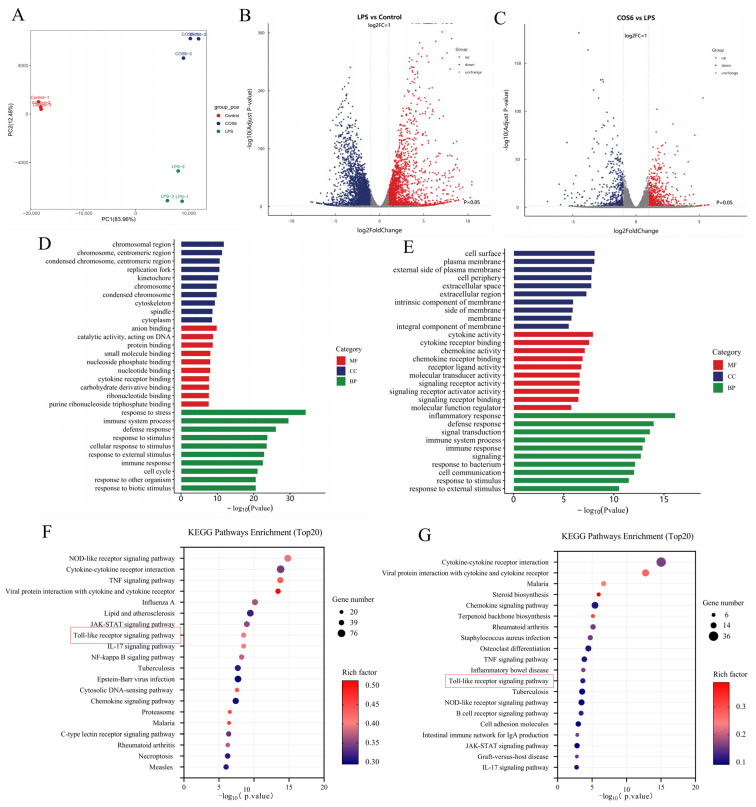
Transcriptome analysis plots of DEGs. (**A**) Two-dimensional principal component analysis (PCA) plot with different colors representing different sample sets. (**B**) Volcano figure of DEGs between the LPS-treated group and the Ctrl group, with each dot representing an individual gene. The gray area signifies no significant difference between the two groups; red dots indicate prominent significantly upregulated genes; and blue dots represent significantly down-regulated genes (|log2FC| > 1 and *p*-value < 0.05); (**C**) Volcanic images of DEGs between COS6 and LPS. (**D**) Histogram of GO enrichment of differentially expressed genes for COS6 vs. LPS. GO describes the properties of genes and their products in organisms in terms of Biological Process (BP), Molecular Function (MF), as well as Cellular Component (CC). In this enrichment histogram showing 30 functional traits with high DEG enrichment, the vertical axis shows different GO terms and the horizontal axis shows *p*-values. (**E**) Histogram of GO enrichment of DEGs for COS6 vs. LPS. (**F**) KEGG enrichment analysis of LPS vs. Ctrl upregulated DEGs. The bubble plot shows 20 pathways with high DEG enrichment; the size of each dot corresponds to the number of DEGs enriched in the counterpart terms. The vertical axis shows different path names, the horizontal axis represents *p*-value, and the color of the dots indicates rich factor. The rich factor represents the ratio of DEGs in a pathway to the total number of genes in that pathway entry across all reference annotation genes; a higher rich factor suggests a higher degree of enrichment, whereas a lower *p*-value denotes more significant enrichment. (**G**) KEGG enrichment analysis bubble plot of COS6 vs. LPS downregulated DEGs.

To elucidate the anti-inflammatory mechanism of COS6 on LPS-stimulated RAW 264.7 macrophages, the anti-inflammatory effect of COS6 was analyzed via an RNA-seq technique. The gene expression levels of COS6 and LPS or LPS and Ctrl were compared to identify differentially expressed genes (DEGs) resulting from COS6 treatment in LPS-stimulated macrophages. Principal component analysis (PCA) (Figure 3A) revealed clear separation among the COS6 group, LPS group, and control group, indicating that COS6 treatment significantly altered gene expression in LPS-stimulated macrophages. Differential gene expression was visualized using volcano plots (Figure 3B,C). A total of 1179 DEGs were identified in COS6-treated LPS-stimulated macrophages (*p* < 0.05), with 711 genes being upregulated and 468 genes downregulated.

GO functional enrichment and KEGG pathway enrichment analysis were investigated to identify inflammatory-related signaling pathway affected by COS6 treatment. The result indicated that DEGs in COS6-treated cells were predominantly associated with biological processes, including inflammatory response, immune response, and signal transduction (Figure 3D,E). KEGG pathway enrichment revealed significant enrichment in pathways including the Toll-like receptor signaling pathway, TNF signaling pathway, NOD-like receptor signaling pathway, and JAK-STAT signaling pathway, all of which were associated with the inhibition of LPS-stimulated inflammatory signaling (Figure 3F,G). RT-qPCR analysis of inflammatory genes showed that COS6 significantly reduced the overexpression of iNOS (Figure 2E), TNF-α (Figure 2F), IL-6 (Figure 2G), and IL-1β (Figure 2H) in LPS-induced macrophages. These results were consistent with the transcriptome data, further confirming that COS6 effectively inhibited the overexpression of multiple inflammation-related genes in LPS-stimulated RAW 264.7 macrophages. Transcriptomic analysis revealed that COS6 inhibited the upstream TLR2 receptor signaling pathway and downstream inflammatory factors, including Tnfrsf1b, Tnfaip3, Il6 (IL-6), Il1b (IL-1β), and Ccl2 (MCP-1), which are part of the NF-κB signaling pathway. Additionally, COS1–7 regulated the overexpression of IL-6 and TNF-α, as well as IL-1β and MCP-1 in LPS-induced macrophages, to varying extents (Figure 2). The NF-κB pathway, controlling the release of these factors, was downstream of the Toll-like receptor signaling pathway [35], TNF pathway [38], and JAK-STAT pathway [39] in the KEGG enriched pathways (Figure 3F). Therefore, we further investigated whether COSs affected the level of key factors in the NF-κB signaling pathway mediated by TLR2 to clarify the anti-inflammatory mechanism of COSs in LPS-stimulated macrophages.

### 2.4. The Anti-Inflammatory Effect of COS on LPS-Stimulated RAW 264.7 Macrophages Through the TLR2/NF-κB Pathway

**Figure 4:**
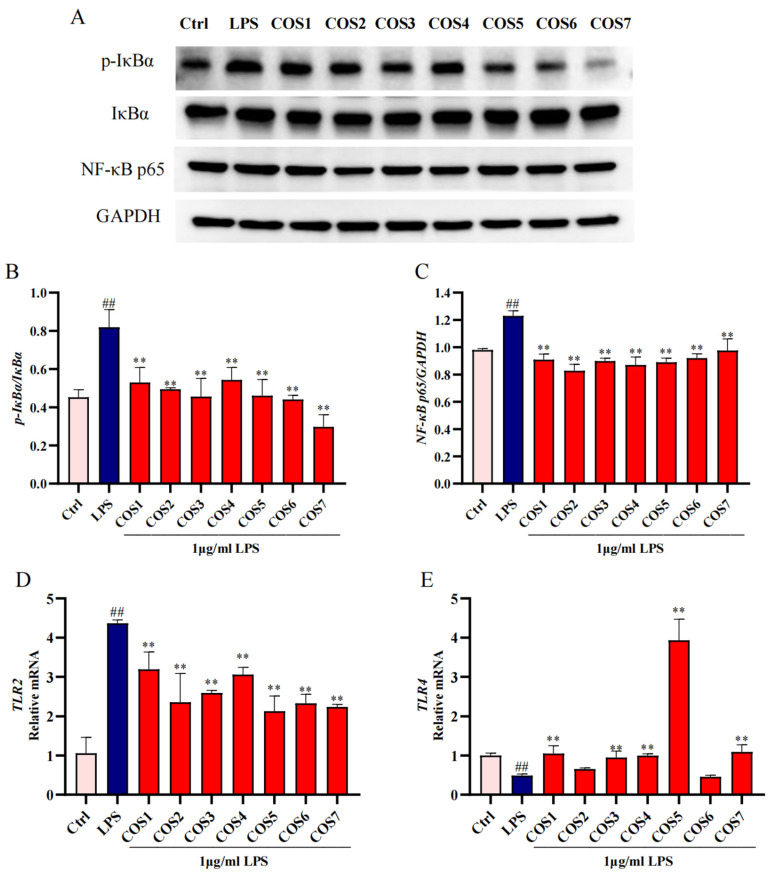
COS1–7 may inhibit LPS-stimulated inflammatory signaling in macrophages through the TLR2/NF-κB pathway. (**A**) The levels of NF-κB proteins in macrophages were measured by western blot, with GAPDH serving as the loading control. (**B**) Quantification of p-IκBα, IκBα proteins, normalized to GAPDH (**C**) Quantification of NF-κB p65 proteins, normalized to GAPDH. mRNA expression levels of (**D**) TLR2 and (**E**) TLR4 were analyzed by RT-qPCR. Relative to Ctrl, ## *p* < 0.01; Relative to LPS, ** *p* < 0.01.

To validate the mechanism by which COSs inhibited LPS-stimulated macrophage inflammatory signaling, we employed Western blot and RT-qPCR to examine the Toll like receptor signaling pathway transduction (Figure 4). COS1–7 significantly (*p* < 0.01) inhibited the overexpression of p-IκBα in RAW 264.7 macrophages stimulated by LPS (Figure 4A,B). Within the polymerization degree range of 1–3 and 4–7, the inhibition effect of COS increased with high degrees of polymerization. For example, COS7 showed the most potent inhibition of p-IκBα expression. Additionally, COSs significantly (*p* < 0.01) inhibited the overexpression of the p65 subunit of NF-κB induced by LPS stimulation (Figure 4A,C). COS2 exhibited the strongest inhibitory effects on p65 expression. Therefore, the inhibition of p-IκBα and p65 by COS1–7 indicated that COSs can regulate downstream cytokines by blocking the NF-κB signaling pathway, thereby exerting anti-inflammatory properties.

An RT-qPCR analysis of TLR2 (Figure 4D) and TLR4 (Figure 4E) mRNA expression revealed that COS1–7 significantly (*p* < 0.01) inhibited the mRNA expression of TLR2, which may have resulted in the inhibition of its downstream signaling pathway. COS5 exhibited the strongest inhibitory effect on TLR2 expression. Except COS2 and COS6, COSs can activate TLR4 mRNA expression. In this regard, COS5 showed the best activating effect (*p* < 0.01).

### 2.5. TLR2 and TLR4 Involved in COS Regulation of LPS-Stimulated Inflammatory Signaling

**Figure 5:**
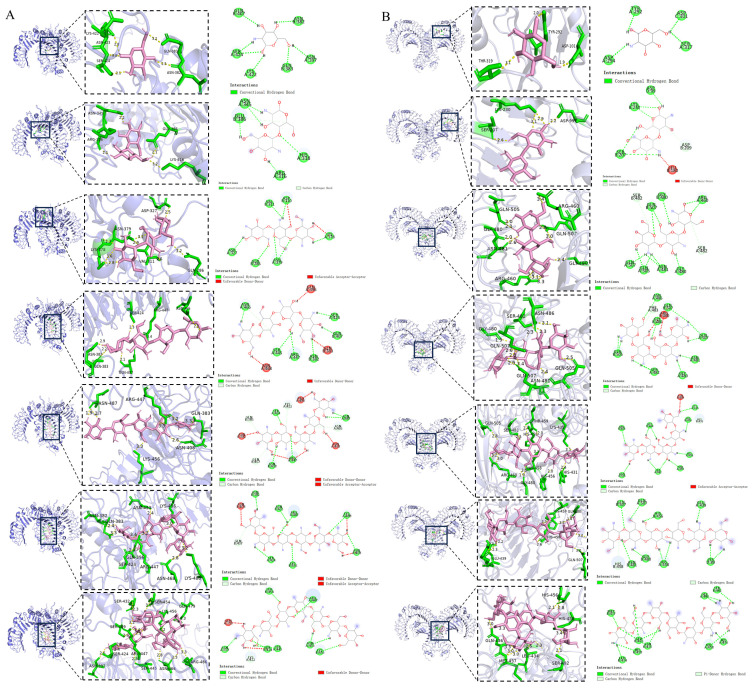
The binding mode of the unit structure of COS1–7 with (**A**) TLR2 and (**B**) TLR4.

The mechanism of COS inhibiting cytokine release induced by LPS was analyzed. A molecular docking analysis was performed to verify whether the anti-inflammatory properties of COS varied with different degrees of polymerization on RAW 264.7 macrophages induced by LPS as a function of the binding affinity and binding sites of TLR2 or TLR4. A binding energy below −5.0 kcal/mol suggested favorable binding and good affinity between the ligand and receptor [40]. COS1–7 were able to bind to both TLR2 and TLR4, and the binding energies were all less than −5 kal/mol, indicating that COS1–7 could interact with TLR2 and TLR4 with sufficient affinity (Table 1 and Table 2). The binding affinity of COS to TLR2 and TLR4 increased from DP 1 to 5 and then decreased from DP 5 to 7. The strongest binding energy value was found with COS5 (−8.5 kal/mol for TLR2, −8.8 kal/mol for TLR4), followed by COS4 (−8.0 kal/mol for TLR2, −8.4 kal/mol for TLR4) and COS6 (−7.8 kal/mol for TLR2, −8.2 kal/mol for TLR4). Figure 5 shows that COS1–7 primarily interacted with the extracellular regions of TLR2 and TLR4 via hydrogen bonds. The number of hydrogen bonds and interacting residues varied depending on the DP.

## 3. Discussion

LPS, acting as an endotoxin, can activate macrophages and induce them to secrete pro-inflammatory cytokines, thereby generating inflammation [41]. It is well-known that IL-6, TNF-α, and IL-1β are associated with inflammation [42]. In addition, as a member of the chemokine family, MCP-1 is vital in innate immunity, attracting monocytes to the site of inflammation [43]. In this study, COS substances with different DP showed significant ability to inhibit LPS-induced NO release, the mRNA expression of iNOS, IL-6, TNF-α, and IL-1β, as well as the secretion levels of IL-6, TNF-α, and MCP-1 in cells (Figure 2). Comparing with other DP values, COS6 showed a prominent and stable inhibitory effect on NO release and the content and mRNA expression of cytokines induced by LPS (Figure 2), identifying COS6 as the most effective anti-inflammatory chitooligosaccharide among the tested COSs. The results indicated that the DP of COSs plays a key role in determining the anti-inflammatory effects induced by LPS. Similar trends were also found in other research works. For example, it was found that for galactose oligosaccharides (GOS, DP 1–4), higher polymerization degrees correlated with stronger anti-inflammatory activity, with GOS4 exhibiting the strongest inhibitory effect on the levels of NO, IL-1β, TNF-α, and IL-6 [44]. Among agaro-oligosaccharides (DP from 2 to 10), neoagarotetraose (DP 4) showed the strongest anti-inflammatory effect by reducing the LPS-induced overexpression of IL-1β, TNF-α, IL-6, and iNOS through down-regulating the MAPK and NF-κB signaling pathways [45]. In the present study, although COS with DP from 1 to 7 showed less potent anti-inflammatory effects compared to classical drugs (dexamethasone [46], ibuprofen [47], and aspirin [48]), their food origin presented a safety advantage for potential dietary applications. This highlights the substantial potential of COSs as promising candidates for anti-inflammatory therapeutic applications.

Transcriptomic analysis has been used to explore the molecular mechanisms of bioactive compounds in exerting anti-inflammatory effects [49,50]. Our RNA-seq study revealed that COS6 exerted its anti-inflammatory effects on LPS-stimulated RAW 264.7 macrophages by down-regulating the expression of receptors such as TLR2, cytokine receptors, and N nucleotide-binding oligouclization domain (NOD)-like receptors (NLRs), which are located upstream of NF-κB signaling pathway. NF-κB, which consists primarily of p50 and p65 subunits, is rendered inactive through its association with inhibitory protein lκBα. Upon extracellular inflammatory stimulation, IκBα is phosphorylated, releasing NF-κB, which translocates rapidly to the nucleus to induce the transcription of target genes, ultimately leading to inflammation [51]. Previous studies found that COS6-mediated NF-κB activation through mannose receptor targeting in an immunomodulatory model [37]. However, our results indicated that COS6 treatment significantly reduced p-IκBα and p65 protein levels while differentially regulating TLRs expression, i.e., suppressing TLR2 while increasing the TLR4 mRNA levels in LPS-induced macrophages (Figure 4). The different receptor recognitions and modulation patterns might have been due to the model variations.

Molecular docking involves spatial and energy matching between ligand and receptor to find the best conformation [52]. Previous studies have identified key residues, such as LYS89 [53], GLN436, and GLU439 [28], involved in LPS binding to TLR4 and the activation of NF-κB, and have also determined that the TLR4 dimerized domain contains amino acids from GLU26 to LYS631 [53]. Additionally, other studies have indicated that ASP310, VAL311, HIS318, ASN345, LYS378, and GLN383 are key residues involved in TLR2 dimerization, whereby the dimerization process of TLR2 initiates with extracellular interactions (from GLN25 to ILE506) [30], which then triggers conformational changes leading to intracellular Toll IL-1 Receptor (TIR) domain dimerization and the activation of the TLR signaling cascade [30]. After the dimerization of TLR2 and TLR4, the TIR domain recruits the adaptor protein myeloid differentiation Factor 88 (MyD88), which activates the IκB kinase, leading to the activation of NF-κB and ultimately increasing the expression of pro-inflammatory cytokines [54]. Our results revealed that COS6 specifically bound to key residues (LYS89, GLN436, and GLU439) in the TLR4 binding pocket, thereby competitively inhibiting LPS-induced TLR4 signaling activation. Furthermore, COS6 demonstrated extensive interactions with nine binding sites (ASN382-LYS488) in the extracellular C-terminal domain of TLR2 (Figure 5), effectively blocking TLR2 dimerization. This dual-receptor targeting mechanism could explain why COS6 exhibited the most potent anti-inflammatory effects among the tested COS compounds in LPS-stimulated macrophages, despite showing weaker binding affinity to TLRs compared to COS5 (Table 1 and Table 2).

In this study, we employed molecular docking to predict and analyze the interactions between COS and TLR2/4. However, we are also aware of the potential limitations of this approach, which may have affected the accuracy of the predicted results. Therefore, our discussion is combined with the RT-qPCR results for comparative validation to minimize the impact of these limitations on the outcomes. These findings indicate that DP affects the interaction sites and binding strength between COS and TLR2/4, influencing receptor dimerization and recognition of LPS, thereby affecting the downstream signaling cascade. However, our study on the relationship between the polymerization degree of COS and receptor-mediated anti-inflammatory effects was conducted at the cellular level, and further exploration at the animal level is still warranted.

## 4. Materials and Methods

### 4.1. Materials and Chemicals

Glucosamine was procured from Yuanye Biotechnology Co., Ltd. (Shanghai, China), and chitooligosaccharides (chitobiose, chitotriose, chitotetraose, chitopentaose, chitohexaose, chitoheptaose) were obtained from Qingdao BZ Oligo Biotech Co., Ltd. (Qingdao, China). Lipopolysaccharide (LPS, *Escherichia coli* 055:B5) was purchased from Solarbio Technology Co., Ltd. (Beijing, China). Dulbecco’s modified Eagle’s medium (DMEM) and fetal bovine serum (FBS) were purchased from Gibco (Waltham, MA, USA). The Cell Counting Kit-8 (CCK-8) and Cell Proliferation Toxicity Assay Kit were procured from New Cell & Molecular Biotech Co., Ltd. (Suzhou, China). Griess reagent kits were sourced from Beyontime Biotechnology Co., Ltd. (Shanghai, China). Mouse ELISA Kits for IL-6 (Cat. No. ab222503), TNF-α (Cat. No ab208348), and MCP-1 (Cat. No ab208979) were obtained from Abcam Trading Co., Ltd. (Cambrige, MA, USA). GAPDH (Cat. No. A19056) was used at a concentration of 1:50,000, IκBα (Cat. No. A19741) at a concentration of 1:1000, phospho-IκBα (Cat. No. AP1294) at a concentration of 1:500, and NF-κB p65 antibodies (Cat. No. A19653) at a concentration of 1:5000. These antibodies were purchased from ABclonal (Wuhan, China). The horse radish peroxidase-conjugated Goat Anti-Rabbit IgG, used at a concentration of 1:50,000, was acquired from Jackson ImmunoResearch (West Grove, PA, USA).

### 4.2. Cell Culture and COS Treatment

The RAW 264.7 macrophage cell line, derived from the ascites of a tumor induced by Abelson murine leukemia virus in BALB/c mice, was purchased from the cell bank of the Chinese Academy of Sciences (Shanghai, China). The cells were maintained within a maximum of 30 passages to ensure phenotypic and functional stability. To monitor the cell morphology and ensure the cells were free from contamination and consistent throughout the experiments, we regularly observed the cells using an inverted microscope. The cells were cultured in DMEM with 10% FBS and incubated at 37 °C in a humidified atmosphere containing 5% CO_2_. Log-phase cells were seeded into either 96-well or 6-well plates and cultured for 24 h to reach a density of 5 × 10^5^ cells per well. Subsequently, the culture media were replaced with 200 μM of COS, and the cells were incubated for an additional 24 h. LPS stimulation was then performed by adding 1 μg/mL of LPS, followed by incubation for either 6 or 24 h. The supernatant and cells were collected for further assays. Untreated cells (no COS and LPS) were used as the Control (Ctrl).

### 4.3. Cell Viability

The effects of COSs on the viability of RAW 264.7 macrophages were assessed using the CCK-8 assay. Briefly, RAW 264.7 macrophages were plated in 96-well plates at a density of 5 × 10^5^ cells per well and incubated for 24 h. Then, 100 μL of COSs at varying concentrations (0–400 μM) was added to the cells and incubated for an additional 24 h. Following this incubation, 10 μL of CCK-8 reagent was added to each well, followed by further incubation for 1 h at 37 °C. Subsequently, the absorbance at 450 nm was measured to determine cell viability.

### 4.4. Griess Reaction and ELISA Kit Assay

The levels of nitrite in the cell culture supernatant secreted after 24 h of LPS stimulation were measured using the Griess assay reagent kit to indirectly infer the production of NO. The levels of cytokines (IL-6, TNF-α, MCP-1) in the supernatant were determined using Mouse ELISA Kits according to the manufacturer’s instructions.

### 4.5. Real-Time Quantitative PCR (RT-qPCR)

The mRNA levels of the iNOS gene, cytokines genes (IL-6, TNF-α, IL-1β), and cell receptors genes (TLR2, TLR4) of RAW 264.7 macrophages after 6h LPS stimulation were determined by RT-qPCR. Briefly, Total RNA of RAW 264.7 macrophages after 6 h LPS stimulation from Section 4.2 was extracted using the RNAsimple Total RNA Kit (TIANGEN, Beijing, China). The total RNA was then reverse transcribed with SynScript^®^ III RT SuperMix (Tsingke, Beijing, China), according to the manufacturer’s protocol. The primers for cDNA detection, which were used in RT-qPCR, are listed in Table 3. The efficiency and specificity of each primer pair were evaluated through melting curve analysis, showing single, sharp peaks for each amplicon, indicating the absence of primer dimers. The amplification curves were also examined to ensure consistent and reliable amplification across three biological replicates. The RT-qPCR reaction was performed using an SYBR qPCR Mix kit (Tsingke, Beijing, China), with an initial denaturation at 95 °C for 1 min, followed by 40 cycles of 95 °C for 10 s and 60 °C for 20 s. The expression levels of the target genes were normalized to β-actin and quantified using the 2^−ΔΔCT^ method.

### 4.6. RNA-Seq

Cells underwent various treatments, as described in Section 4.2. Total RNA was extracted from treated the RAW 264.7 macrophages using Trizol Reagent (Themo Fisher Scientific, Waltham, MA, USA). RNA quality was verified with a Nanodrop ND-2000 system (Thermo Scientific, USA) and Agilent Bioanalyzer 4150 system (Agilent Technologies, Santa Clara, CA, USA). For the library construction, a mRNA-seq Lib Prep Kit (ABclonal, Wuhan, China) was employed according to the manufacturer’s protocol, and sequencing was performed with a MGISEQ-T7 instrument. In simple terms, processing using in-house Perl scripts for the raw sequencing data was applied to remove the adaptor sequences and filter out the low-quality reads. Clean reads then were aligned to a reference genome with orientation mode utilizing HISAT2 software (http://daehwankimlab.github.io/hisat2/, accessed on 1 August 2024, version 2.2.1) to generate mapped reads. Differential expression analysis was performed using DESeq2 (http://bioconductor.org/packages/release/bioc/html/DESeq2.html, accessed on 1 August 2024, version 1.34.0); genes with |log2FC| > 1 and *p*-value < 0.05 were considered significantly differentially expressed genes (DEGs) between the LPS and control group or the COS6 and LPS group. Finally, we used the clusterProfiler software package (v 4.16) for Gene Ontology (GO) function enrichment as well as Kyoto Encyclopedia of Genes and Genomes (KEGG) pathway enrichment analysis. A *p*-value < 0.05 indicated significant enrichment of GO or KEGG function; the differences between samples were clarified at the level of gene function.

### 4.7. Western Blot

After 6 h of LPS stimulation (as detailed in Section 4.5), cells were harvested and lysed in ice-cold RIPA buffer (Beyotime Biotechnology, Shanghai, China) containing 1 × protease/phosphatase inhibitors. Whole-cell protein extracts were subsequently prepared through centrifugation at 4 °C (12,000× *g*, 10 min). The total protein concentration was determined using a BCA protein assay kit (Beyotime Biotechnology, Shanghai, China). Proteins were separated by SDS-PAGE electrophoresis and transferred to PVDF membranes (Millipore, Waltham, MA, USA). Subsequently, the PVDF membranes were blocked with 5% skim milk at room temperature for 1 h. Rabbit anti-mouse primary antibodies, including NF-κB p65, IκBα, and phospho-IκBα (p-IκBα), were used for immunoblotting. Horseradish peroxidase conjugated goat anti-rabbit IgG was used as a secondary antibody. Finally, the protein bands were visualized using BeyoECL Plus (Beyontime Biotechnology, Shanghai, China) and imaged using the Azure 600 system, and the grayscale values were analyzed using ImageJ software (v1.54i).

### 4.8. Molecular Docking

The structures of TLR2 (PDB ID: 2Z7X) and TLR4 (PDB ID: 3FXI) were obtained from a protein database (www.rcsb.org, accessed on 1 July 2024). Solvents, ligands, and ions were removed from the structures using PyMOL 3.0.3 software. COS1 (Compound ID: 9859210), COS2 (Compound ID: 57369765), COS3 (Compound ID: 70702331), COS4 (Compound ID: 70702329), COS5 (Compound ID: 170920307), COS6 (Compound ID: 92132215), and COS7 (Compound ID: 102602304) were sourced from the PubChem database (https://pubchem.ncbi.nlm.nih.gov/, accessed on 1 June 2024). Molecular docking simulations were conducted using AutoDock vina 1.1.2 to predict potential binding sites between COS 1–7 and TLR4 or TLR2 [55]. For each target protein, a grid box was defined to cover the entire binding site. The grid box for TLR2 was centered at defined coordinates (−27.294, −8.146, 23.008) with dimensions of 20.078 Å × 120.078 Å × 116.266 Å. For TLR4, the grid box was centered at defined coordinates (11.138, −3.85, 0.136) with dimensions of 126.0 Å × 126.0 Å × 126.0 Å. The docking process was repeated nine times to ensure the consistency and reliability of the results. The binding pose with the lowest docking energy was select as the most favorable binding model. The molecular docking results were further visualized and analyzed using PyMOL 3.0.3 and Discovery studio 2019 software.

### 4.9. Statistical Analysis

Results are expressed as standard deviation (SD) ± mean (*n* = 3). Statistically significance was determined using one-way analysis of variance and Duncan test in GraphPad Prism 9.5.1. Differences with *p*-value less than 0.05 or 0.01 were considered statistically significant.

## 5. Conclusions

The results indicated that COS1–7 inhibit the activation of the downstream NF-κB signaling pathway by regulating TLR2/4 levels on the surface of macrophages in response to LPS, which, in turn, reduces the expression of NO, IL-6, TNF-α, MCP-1, and as IL-1β. The inhibitory effect is closely related to DP, which influences the interaction sites and the affinity between COS and TLR2/4, thereby affecting receptor dimerization and LPS recognition. COS6 exhibits the strongest inhibitory effect on the downstream cytokines in LPS-stimulated macrophages by down-regulating TLR2 expression. Molecular docking confirmed that COS6 inhibits TLR2 dimerization and prevents TLR4 recognition of LPS. These findings provide deep insights into the anti-inflammatory mechanisms of COSs via receptor-mediated signaling pathways.

## Figures and Tables

**Table 1:** Binding energy and binding sites of COS1–7 and TLR2.

Sample Name	TLR2 Binding Energy (kal/mol)	Potential Binding Sites
COS1	−5.8	ASN382; GLN383; ASN397; GLN407; LYS422; SER424
COS2	−6.0	ARG316; HIS318; ASN345; GLU386
COS3	−6.0	GLN286; ASP310; VAL311; ASP327; LYS378; ASN379
COS4	−8.0	ASN382; GLN383; GLN396; GLN407; SER424; SER445; ARG447; ASN466; ASN468
COS5	−8.5	ASN382; GLN383; GLN407; ASN408; LYS422; SER424; ARG447; ASN466; ASN468; ARG486; ASN487
COS6	−7.8	ASN382; GLN407; ASN408; SER424; ASN433; ARG447; LYS456; ASN468; LYS488
COS7	−7.3	GLN396; ASN397; LYS422; SER424; SER445; ARG447; SER454; LYS456; ASN466

**Table 2:** Binding energy and binding sites of COS1–7 and TLR4.

Sample Name	TLR4 Binding Energy (kal/mol)	Potential Binding Sites
COS1	−5.6	ASP101; TYR292; ASP294; SER317
COS2	−6.8	ASP99; LEU180; SER207; ASP209; LYS230
COS3	−7.8	GLY480; ASN481; SER482; ARG460; GLN505; GLN507
COS4	−8.4	ARG460; GLY480; ASN481; SER482; PHE483; ASN486; GLN505; GLN507
COS5	−8.8	ASN409; HIS431; LYS435; HIS456; HIS458; THR459; ARG460; GLY480; SER482; GLN505
COS6	−8.2	LYS89; GLU439; HIS456; HIS458; GLY480; ASN481; GLN507
COS7	−7.5	LEU434; GLN436; MET437; GLU439; HIS456; GLY480; SER482; HIS458

**Table 3:** Sequences of primers used in the gene expression analysis.

Genes	Forward Primer (5′–3′)	Reverse Primer (5′–3′)
β-actin	CTAGGCGGACTGTTACTGAGC	ATGTTTGCTCCAACCAACTGC
iNOS	GTCCGAAGCAAACATCACATTCA	GGTAAACACGTTCTTTGCATGGA
IL-6	GTATGAACAACGATGATGCACTTG	CTCTCTGAAGGACTCTGGCTTTG
TNF-α	GACCCTCACACTCAGATCATCTT	CCTTGAAGAGAACCTGGGAGTAG
IL-1β	CAGCACATCAACAAGAGCTTCAG	GAGGATGGGCTCTTCTTCAAAGA
TLR2	GGAGATGTGTCCGCAATCATAGT	CCAAGATCCAGAAGAGCCAAAGA
TLR4	TCCCTGCATAGAGGTGTGAAATT	CCACAGCCACCAGATTCTCTAAA

## Data Availability

The data presented in this study are available on request from the corresponding author.

## References

[1] Hou C., Chen L., Yang L., Ji X. (2020). An insight into anti-inflammatory effects of natural polysaccharides. Int. J. Biol. Macromol..

[2] Barnes P.J. (2017). Cellular and molecular mechanisms of asthma and COPD. Clin. Sci..

[3] Zhang Q., Dehaini D., Zhang Y., Zhou J., Chen X., Zhang L., Fang R.H., Gao W., Zhang L. (2018). Neutrophil membrane-coated nanoparticles inhibit synovial inflammation and alleviate joint damage in inflammatory arthritis. Nat. Nanotechnol..

[4] Bjorkegren J., Lusis A.J. (2022). Atherosclerosis: Recent developments. Cell.

[5] Li D.T., Feng Y., Tian M.L., Ji J.F., Hu X.S., Chen F. (2021). Gut microbiota-derived inosine from dietary barley leaf supplementation attenuates colitis through PPAR gamma signaling activation. Microbiome.

[6] Biffi G., Tuveson D.A. (2021). Diversity and biology of cancer-associated fibroblasts. Physiol. Rev..

[7] Huynh N.C., Nguyen T.T.T., Nguyen D.T.C., Tran T.V. (2023). Occurrence, toxicity, impact and removal of selected non-steroidal anti-inflammatory drugs (NSAIDs): A review. Sci. Total Environ..

[8] Fatima M., Mir S., Ali M., Hassan S., Ul Haq Khan Z., Waqar K. (2024). Synthesis and applications of chitosan derivatives in food preservation-A review. Eur. Polym. J..

[9] Kumari R., Kumar M., Vivekanand V., Pareek N. (2023). Chitin biorefinery: A narrative and prophecy of crustacean shell waste sustainable transformation into bioactives and renewable energy. Renew. Sustain. Energy Rev..

[10] Liaqat F., Eltem R. (2018). Chitooligosaccharides and their biological activities: A comprehensive review. Carbohydr. Polym..

[11] Pan Z., Cheng D., Wei X., Li S., Guo H., Yang Q. (2021). Chitooligosaccharides inhibit tumor progression and induce autophagy through the activation of the p53/mTOR pathway in osteosarcoma. Carbohydr. Polym..

[12] Laokuldilok T., Potivas T., Kanha N., Surawang S., Seesuriyachan P., Wangtueai S., Phimolsiripol Y., Regenstein J.M. (2017). Physicochemical, antioxidant, and antimicrobial properties of chitooligosaccharides produced using three different enzyme treatments. Food Biosci..

[13] Chotphruethipong L., Chanvorachote P., Reudhabibadh R., Singh A., Benjakul S., Roytrakul S., Hutamekalin P. (2023). Chitooligosaccharide from Pacific White Shrimp Shell Chitosan Ameliorates Inflammation and Oxidative Stress via NF-κB, Erk1/2, Akt and Nrf2/HO-1 Pathways in LPS-Induced RAW264.7 Macrophage Cells. Foods.

[14] Ouyang A., Zhang M., Yuan G., Liu X., Su J. (2023). Chitooligosaccharide boosts the immunity of immunosuppressed blunt snout bream against bacterial infections. Int. J. Biol. Macromol..

[15] Fang Z., Cong W., Zhou H., Zhang J., Wang M. (2024). Biological activities, mechanisms and applications of chitooligosaccharides in the food industry. J. Funct. Foods.

[16] Pangestuti R., Bak S., Kim S. (2011). Attenuation of pro-inflammatory mediators in LPS-stimulated BV2 microglia by chitooligosaccharides via the MAPK signaling pathway. Int. J. Biol. Macromol..

[17] Hao W.T., Li K.C., Ge X.Y., Yang H.Y., Xu C.J., Liu S., Yu H.H., Li P.C., Xing R.G. (2022). The Effect of N-Acetylation on the Anti-Inflammatory Activity of Chitooligosaccharides and Its Potential for Relieving Endotoxemia. Int. J. Mol. Sci..

[18] Chang S., Lin Y., Wu G., Huang C., Tsai G.J. (2019). Effect of chitosan molecular weight on anti-inflammatory activity in the RAW 264.7 macrophage model. Int. J. Biol. Macromol..

[19] Liang T.W., Chen W.T., Lin Z.H., Kuo Y.H., Nguyen A.D., Pan P.S., Wang S.L. (2016). An Amphiprotic Novel Chitosanase from Bacillus mycoides and Its Application in the Production of Chitooligomers with Their Antioxidant and Anti-Inflammatory Evaluation. Int. J. Mol. Sci..

[20] Ma P., Liu H., Wei P., Xu Q., Bai X., Du Y., Yu C. (2011). Chitosan oligosaccharides inhibit LPS-induced over-expression of IL-6 and TNF-α in RAW264.7 macrophage cells through blockade of mitogen-activated protein kinase (MAPK) and PI3K/Akt signaling pathways. Carbohydr. Polym..

[21] Qiao Y., Ruan Y., Xiong C., Xu Q., Wei P., Ma P., Bai X., Du Y. (2010). Chitosan oligosaccharides suppressant LPS binding to TLR4/MD-2 receptor complex. Carbohydr. Polym..

[22] Erridge C., Bennett-Guerrero E., Poxton I.R. (2002). Structure and function of lipopolysaccharides. Microbes Infect..

[23] Fuke N., Nagata N., Suganuma H., Ota T. (2019). Regulation of Gut Microbiota and Metabolic Endotoxemia with Dietary Factors. Nutrients.

[24] Gorabi A.M., Kiaie N., Khosrojerdi A., Jamialahmadi T., Al-Rasadi K., Johnston T.P., Sahebkar A. (2022). Implications for the role of lipopolysaccharide in the development of atherosclerosis. Trends Cardiovasc. Med..

[25] Hersoug L.G., Moller P., Loft S. (2016). Gut microbiota-derived lipopolysaccharide uptake and trafficking to adipose tissue: Implications for inflammation and obesity. Obes. Rev..

[26] Zhang X., Tian X., Wang Y., Yan Y., Wang Y., Su M., Lv H., Li K., Hao X., Xing X. (2024). Application of lipopolysaccharide in establishing inflammatory models. Int. J. Biol. Macromol..

[27] Triantafilou M., Triantafilou K. (2002). Lipopolysaccharide recognition: CD14, TLRs and the LPS-activation cluster. Trends Immunol..

[28] Park B.S., Song D.H., Kim H.M., Choi B.S., Lee H., Lee J.O. (2009). The structural basis of lipopolysaccharide recognition by the TLR4-MD-2 complex. Nature.

[29] Yang R.B., Mark M.R., Gray A., Huang A., Xie M.H., Zhang M., Goddard A., Wood W.I., Gurney A.L., Godowski P.J. (1998). Toll-like receptor-2 mediates lipopolysaccharide-induced cellular signalling. Nature.

[30] Jin M.S., Kim S.E., Heo J.Y., Lee M.E., Kim H.M., Paik S., Lee H., Lee J. (2007). Crystal Structure of the TLR1-TLR2 Heterodimer Induced by Binding of a Tri-Acylated Lipopeptide. Cell.

[31] Jin Y., Nguyen T.L.L., Myung C., Heo K. (2022). Ginsenoside Rh1 protects human endothelial cells against lipopolysaccharide-induced inflammatory injury through inhibiting TLR2/4-mediated STAT3, NF-κB, and ER stress signaling pathways. Life Sci..

[32] Zhang B., Liang J., Fan H., Lei K., Li H., Liu D., Zheng F., He M., Chen Y. (2024). Study on anti-inflammatory effect of Shangkehuangshui in vitro and in vivo based on TLR4/TLR2-NF-κB signaling pathway. J. Ethnopharmacol..

[33] Asanka Sanjeewa K.K., Jayawardena T.U., Kim H., Kim S., Shanura Fernando I.P., Wang L., Abetunga D.T.U., Kim W., Lee D., Jeon Y. (2019). Fucoidan isolated from Padina commersonii inhibit LPS-induced inflammation in macrophages blocking TLR/NF-κB signal pathway. Carbohydr. Polym..

[34] Mao N., Yu Y., Lu X., Yang Y., Liu Z., Wang D. (2024). Preventive effects of matrine on LPS-induced inflammation in RAW 264.7 cells and intestinal damage in mice through the TLR4/NF-κB/MAPK pathway. Int. Immunopharmacol..

[35] Chen J., Szodoray P., Zeher M. (2016). Toll-Like Receptor Pathways in Autoimmune Diseases. Clin. Rev. Allergy Immunol..

[36] Hao W.T., Li K.C., Liu S., Yu H.H., Li P.C., Xing R.E. (2023). Pleiotropic Modulation of Chitooligosaccharides on Inflammatory Signaling in LPS-Induced Macrophages. Polymers.

[37] Chen J., Yang Y., Xu Z., Li F., Yang M., Shi F., Lin L., Qin Z. (2023). Characterization of effects of chitooligosaccharide monomer addition on immunomodulatory activity in macrophages. Food Res. Int..

[38] Hayden M.S., Ghosh S. (2014). Regulation of NF-κB by TNF family cytokines. Semin. Immunol..

[39] Guo Q., Jin Y.Z., Chen X.Y., Ye X.M., Shen X., Lin M.X., Zeng C., Zhou T., Zhang J. (2024). NF-κB in biology and targeted therapy: New insights and translational implications. Signal Transduct. Target. Ther..

[40] Ma M., Chen L., Tang Z., Song Z., Kong X. (2023). Hepatoprotective effect of total flavonoids from *Carthamus tinctorius* L. leaves against carbon tetrachloride-induced chronic liver injury in mice. Fitoterapia.

[41] Di Lorenzo F., Duda K.A., Lanzetta R., Silipo A., De Castro C., Molinaro A. (2022). A Journey from Structure to Function of Bacterial Lipopolysaccharides. Chem. Rev..

[42] He X.M., Liu L.Y., Gu F.L., Huang R.S., Liu L., Nian Y.T., Zhang Y.Y., Song C. (2024). Exploration of the anti-inflammatory, analgesic, and wound healing activities of *Bletilla Striata* polysaccharide. Int. J. Biol. Macromol..

[43] Hu H., Xia H., Zou X., Li X., Zhang Z., Yao X., Yin M., Tian D., Liu H. (2021). N-acetyl-chitooligosaccharide attenuates inflammatory responses by suppression of NF-κB signaling, MAPK and NLRP3 inflammasome in macrophages. J. Funct. Foods.

[44] Sun C.C., Hao B.F., Pang D.R., Li Q., Li E.R., Yang Q., Zou Y.X., Liao S.T., Liu F. (2022). Diverse Galactooligosaccharides Differentially Reduce LPS-Induced Inflammation in Macrophages. Foods.

[45] Wang W., Liu P., Hao C., Wu L., Wan W., Mao X. (2017). Neoagaro-oligosaccharide monomers inhibit inflammation in LPS-stimulated macrophages through suppression of MAPK and NF-κB pathways. Sci. Rep..

[46] Liu H.L., Zhou L., Wang X.F., Zheng Q.C., Zhan F.F., Zhou L.Q., Dong Y., Xiong Y.H., Yi P.C., Xu G.H. (2024). Dexamethasone upregulates macrophage PIEZO1 via SGK1, suppressing inflammation and increasing ROS and apoptosis. Biochem. Pharmacol..

[47] Chen L., Xie L.M., Zhang J., Feng Y.F., Wu X. (2023). GC-MS analysis of fatty acid metabolomics in RAW264.7 cell inflammatory model intervened by non-steroidal anti-inflammatory drugs and a preliminary study on the anti-inflammatory effects of NLRP3 signaling pathway. PLoS ONE.

[48] Liu Y.T., Fang S.L., Li X.Y., Feng J., Du J., Guo L.J., Su Y.Y., Zhou J., Ding G., Bai Y.X. (2017). Aspirin inhibits LPS-induced macrophage activation via the NF-κB pathway. Sci. Rep..

[49] Huo J.Y., Pei W.H., Liu G.Y., Sun W.Z., Wu J.H., Huang M.Q., Lu W., Sun J.Y., Sun B.G. (2023). Huangshui Polysaccharide Exerts Intestinal Barrier Protective Effects through the TLR4/MyD88/NF-κB and MAPK Signaling Pathways in Caco-2 Cells. Foods.

[50] Fu X.D., Huang X.R., Tan H.Z., Huang X.J., Nie S.P. (2024). Regulatory Effect of Fucoidan Hydrolysates on Lipopolysaccharide-Induced Inflammation and Intestinal Barrier Dysfunction in Caco-2 and RAW264.7 Cells Co-Cultures. Foods.

[51] Hoffmann A., Levchenko A., Scott M.L., Baltimore D. (2002). The IκB-NF-κB signaling module: Temporal control and selective gene activation. Science.

[52] Zhang J., Li F., Shen S., Yang Z., Ji X., Wang X., Liao X., Zhang Y. (2023). More simple, efficient and accurate food research promoted by intermolecular interaction approaches: A review. Food Chem..

[53] Kim H.M., Park B.S., Kim J., Kim S.E., Lee J., Oh S.C., Enkhbayar P., Matsushima N., Lee H., Yoo O.J. (2007). Crystal Structure of the TLR4-MD-2 Complex with Bound Endotoxin Antagonist Eritoran. Cell.

[54] Fitzgerald K.A., Kagan J.C. (2020). Toll-like Receptors and the Control of Immunity. Cell.

[55] Trott O., Olson A.J. (2010). Software News and Update AutoDock Vina: Improving the Speed and Accuracy of Docking with a New Scoring Function, Efficient Optimization, and Multithreading. J. Comput. Chem..

